# Supplementary material for: International professional practices in mental health, organization of psychiatric care, and COVID-19: A survey protocol
Source: PLoS One. 2021 Dec 28;16(12):e0261818. doi: 10.1371/journal.pone.0261818 (PMC8714114; doi:10.1371/journal.pone.0261818)
Supplement: S1 File — (PDF) [file pone.0261818.s001.pdf]

Interview grid for individual interviews

Interview grid for individual interviews
**<u>Objectives :</u>**<br>To document and understand the personal experiences of mental health professionals faced with the health crisis linked to the Covid-19 pandemic, the experience they gained from it for their therapeutic practices, their questioning, their point of view on the situation of psychiatry and mental health in their country.<br><br>**<u>Form:</u>**<br>Order of questions mutable to follow the respondent's line of thought and questions to be adjusted according to the respondent's profile. Vigilance point: make sure that the questions are anchored in the reality of the situations encountered, while at the same time leading to a reflexivity about the experiences, to avoid the pitfall of a generic analysis of the health crisis in the respondent's country.
**Theme 1: Local organisational adaptations**
**1.1 The occurrence of the Covid-19 pandemic has probably impacted your professional activity. Can you describe what have been the major changes for you as a (profession) since the outbreak of the epidemic in your country?**<br><br>**1.2 What has this changed for your daily professional practice?**<br><br>**1.3 Do you think that these adaptations have optimally reconciled continuity of care with limiting the risk of transmission of the virus?**<br><br>**1.4 What room for manoeuvre have you had to adapt your work in this new context of health crisis?**<br><br>**1.5 Have you been guided in your choices by good practice guidelines in this new context?**<br><br>**1.6 In your opinion, did the organisational changes implemented meet the ethical principles of your profession?**
**Theme 2 : The use of telepsychiatry and e-mental health monitoring methods.**
**2.1 One of the solutions to limit the transmission of the virus is to carry out activities "remotely" via telephone and computer, in particular by videoconference. Have you been concerned by this transition "from face-to-face to remote"?**<br><br>**2.2 How has this "remote" approach impacted the way you have worked?**<br><br>**2.3 How has this "remote" approach impacted your relations with your colleagues?**<br><br>**2.4 How has this "remote” approach impacted your relationships with patients?**

**2.5 Have you used mobile apps or connected objects in your work?**<br><br>**2.6 Do you think that the use of these new technologies should be further developed in the context of your profession?**
**Theme 3 : The experience of psychiatry at the time of Covid-19, professional and personal experiences**
**3.1 The Covid-19 crisis has had a major impact on working conditions in mental health services in Europe. In relation to your profession, what is special about this impact?**<br><br>**3.2 Personally, how did you experience this period?**<br><br>**3.3 Do you think that your own mental health has been affected?**<br><br>**3.4 Were you afraid of contracting Covid-19 in the course of your work?**<br><br>**3.5 The Covid-19 epidemic has forced the mental health sector to adapt and innovate. What lessons have you learnt from this crisis for your professional practices?**<br><br>**3.6 Do you think that the provision of psychiatric and mental health care in your country was in a favourable position compared to other European countries?**
**Respondent profile**

Interview grid for focus groups

**Interview grid for focus groups**
**Objectives :**<br>To compare the personal experiences of mental health professionals faced with the health crisis linked to the Covid-19 pandemic, their points of view and questions in order to highlight divergences and consensus. In addition to the production of data, the focus groups should contribute to the mutual enrichment of professionals, as well as to the interprofessional construction of an ethical reflexivity and common practical solutions.<br><br>**Form :**<br>For this purpose, it is preferable to set up small groups, bringing together professionals of different status practising in the same country. The facilitator will ensure that the speakers are distributed so that everyone can express themselves.
**Theme 1 : Local organisational adaptations**
**1.1 The occurrence of the Covid-19 pandemic has probably impacted your professional activities. Can you describe what were the major changes?**<br><br>**1.2 Do you think that these adaptations have optimally reconciled the continuity of care and the limitation of the risk of transmission of SARS-Cov-2?**<br><br>**1.3 In your opinion, are these organisational adaptations problematic?**
**Theme 2 : The use of telepsychiatry and e-mental health monitoring methods.**
**2.1 One of the solutions to limit the transmission of the virus is to carry out activities "remotely" by videoconference in particular. Have you been concerned by this transition "from face-to-face to distance"?**<br><br>**2.2 How has the shift of certain activities to "remote" approach video-conferencing impacted the way you have worked?**<br><br>**2.3 Do you think the use of these new technologies should be further developed?**
**Theme 3 : The experience of psychiatry at the time of Covid-19, professional and personal experiences**
**3.1 The Covid-19 crisis has had a major impact on working conditions in mental health services in Europe. How did you experience this period, individually and collectively?**<br><br>**3.2 Do you think that this crisis has affected the mental health of mental health professionals?**

3.3 Do you think that the provision of psychiatric and mental health care in your country was in a favourable position compared to other European countries?

Respondents profile

Questionnaire

Questionnaire

Objectives :
Identify, document and quantify in a global way : the situations and difficulties faced by psychiatric and mental health professionals; their adaptations and recommendations for dealing with them; the organizational and ethical issues involved.
Assess the occupational health of these professionals - using the GHQ (General Health Questionnaire) - and their way of working in mental health - using the MHPCI questionnaire (Mental Health Professional Culture Inventory).

Form :
online questionnaire, self-questionnaire with yes/no questions, multiple choice questions and open-ended questions on the key points of the study, designed to complement individual interviews and focus groups.

Theme 1 : Local organisational adaptations

1.During the Covid-19 pandemic, was your professional activity interrupted ?
(drop down list)

- yes, for a few days
- yes, for a few weeks
- yes, for several months
- no, my professional activity has been maintained

1.a - If yes
During which period ?
(one or more checkboxes)

- between January and June 2020
- between July and December 2020
- between January and June 2021

1.b - Has the interruption of your professional activity been:
(1 check box)

- imposed by law
- decided by the direction of the structure in which you work
- decided by you

1.b.1 - If imposed by law or decided by the direction of the structure in which you work
Did this forced interruption of your professional activity seem to you :
(1 check box)

- justified and adequate
- unjustified and inadequate

1.b.1.bis - Why ?
(open question)

1.b.2 - If decided by you

Why ?
(open question)

1.c - Due to the interruption of your professional activity, have you been subject to:
(drop-down list)

- a slight decrease in your income
- a significant drop in your income
- a significant drop in your income implying financial difficulties
- not concerned, my income was maintained

1.c.1 - If decrease or drop in income
During which period ?
(one or more checkboxes)

- between January and June 2020
- between July and December 2020
- between January and June 2021

1.d - Have you restarted your activity since this interruption ?
(yes/no question)

- yes
- no

1.d.1 - If no
What would be your operational recommendations for the psychiatry and mental health sector in your country ?
(open question)
& end of the questionnaire

1.d. 2 - If yes, we go back to the "normal" questionnaire

2.In order to limit the risk of transmission of SARS-CoV-2, which adaptations have been made in the context of your professional activity ?
(one or more checkboxes)

- the use of personal protective equipment (masks, gloves, gowns)
- the disinfection of surfaces, equipment and premises
- the regular ventilation of the spaces
- the temperature measurement
- the realisation of screening tests on a case-to-case basis
- the systematic realisation of screening tests
- the layout of spaces or units for suspicious cases
- the layout of spaces or units for positive cases
- the reduction of the number of consultations
- the reduction of the number of hospital admissions
- the reduction of home visits
- the reorganization of schedules
- the reconstitution of the teams
- the closing of waiting rooms
- the passage of certain activities from face-to-face to distance (telephone, video-conferencing, etc.)
- other : ...

3.Do you think that these adaptations have made it possible to:
(one or more check boxes)

- limit the risk of SARS-CoV-2 transmission
- ensure continuity of care and support for all your patients
- ensure adequate follow-up for your most vulnerable patients
- provide minimal support to some of your patients
- other : ...

4.Because of the Covid-19 crisis, have you been led to carry out unusual activities ?
(yes/no question)

- yes
- no

<u>4.1 - If yes</u>
Have you worked:
(one or more checkboxes)

- in another department or structure than the one in which you normally work
- within the framework of a program dedicated to psychological care in times of crisis caused by Covid-19
- to support front-line health care professionals
- to support other mental health service professionals
- to support the general population and promote mental well-being
- to support positive psychiatric patients at Covid-19
- to support Covid-19 positive patients with no psychiatric history
- to develop recommendations for care in the context of the Covid-19 pandemic
- other : …

5.In your opinion, did the organizational adaptations implemented meet the ethical and deontological principles inherent to your profession ?
(drop-down list)

- strongly agree
- rather agree
- rather disagree
- disagree at all

6.Do you think that some organizational adaptations have raised ethical or deontological issues ? Which ones ?
(open question)

7.Has the Covid-19 pandemic interrupted the training activities of young professionals in your professional framework ?
(one check box)

- yes
- no
- not relevant (no training activity)

Theme 2 : The use of telepsychiatry and e-psychiatry modalities

8.Since the outbreak of the Covid-19 pandemic, have you used, <u>in the course of your relations **with your colleagues**</u>:
(one or more checkboxes)

- appointments or meetings by video-conferencing

- telephone calls
- e-mail exchanges
- chat or sms exchanges
- none of these terms and conditions
- other : ...

8.a - The professional use of teleconferencing for exchanges with your **colleagues** during the Covid-19 pandemic is for you:
(1 check box)
- a novelty never experienced before
- a practice initiated before the Covid-19 pandemic
- a habit developed before the Covid-19 pandemic

9.Since the outbreak of the Covid-19 pandemic, have you used, for the therapeutic follow-up **of your patients :**
(one or more checkboxes)
- appointments or meetings by video-conferencing
- video-conferencing group therapy sessions
- telephone calls
- e-mail exchanges
- chat or sms exchanges
- none of these terms and conditions
- other : ...

9.a - If checked
The professional use of teleconferencing for the follow-up of your **patients** during the Covid-19 pandemic is for you:
(1 check box)
- a novelty never experienced before
- a practice initiated before the Covid-19 pandemic
- a habit developed before the Covid-19 pandemic

10.How often do you use teleconferencing ?
(drop down list)
- every months
- every week
- every day
- several times a day
- never

10.a - If any box checked except never
What is the name of the teleconferencing platform(s) you have used ?
(open question)

11.In your professional activities, have you used:
(one or more checkboxes)
- your personal computer
- your personal phone
- a personal email account (Skype, Zoom, WhatsApp, other)
- exclusively tools dedicated to your professional activity

12.Have you used mobile apps in the course of your professional activity ?
(for example : HappliDay, Medscape, TousAntiCovid)

(yes/no question)

- yes
- no

12.a - If yes
What was the aim ?
(one or more checkboxes)

- diagnostic assistance
- assessment and treatment assistance
- monitoring of certain parameters in your patients
- therapeutic education of your patients
- cognitive-behavioural therapy tools for your patients
- contact tracking to reduce the risk of SARS-CoV-2 transmission

12.b - What is the name of the mobile apps you have used ?
(open question)

13.Have you used connected objects in the course of your professional activity ?
(for example : t-shirt connected, watch connected, bike connected)
(yes/no question)

- yes
- no

13.a - Si oui

What is the name of connected objects you have used ?
(open question)

14.Does the use of these different technological tools pose technical or practical difficulties for you ? which ones ?
(open question)

15.Does the use of these different technological tools pose ethical or deontological difficulties for you ? which ones ?
(open question)

Theme 3: The experience of psychiatry at the time of Covid-19, professional and personal experience

16.Have you experienced an increase in your workload as a result of the Covid-19 pandemic?
(compared to the usual hourly volume and amount of tasks)
(1 check box)

- yes
- no

16.a - If yes
This increase in your workload was caused by :
(one or more checkboxes)

- an increase in the number of patients
- the increasing complexity of care
- a diversification in your tasks
- an increase in your responsabilities

17.Have you been confronted with new situations in which you did not feel experienced enough?

(1 check box)

- yes
- no

17.a - If yes
17.b - In which situations ?
(open questions)

18. Have you carried out your activity as a teleworker?
(drop down list)

- no, I have not teleworked
- yes, but only for a few weeks
- yes, partially for several months
- yes, totally for several months

18.a - If yes
During which period ?
(one or more checkboxes)

- between January and June 2020
- between July and December 2020
- between January and June 2021

18.b - What advantages have you found in teleworking ?
(open question)

18.c - And what drawbacks did you face?
(open question)

19.Has the Covid-19 crisis made it more difficult to reconcile your professional life with your personal and family life?
(1 check box)

- yes
- no

20.Why ?
(open question)

21.As a result of measures impacting your professional activity, have you been subject to:
(drop down list)

- yes, slight decrease in income
- yes, significant drop in income
- yes, significant drop in income implying financial difficulties
- no

21.a - If yes
During which period ?
(one or more checkboxes)

- between January and June 2020
- between July and December 2020
- between January and June 2021

22.Are you afraid of contracting Covid-19 in the course of your professional activity?
(1 check box)

- yes

- no

23.Have you ever been confronted with Covid-19 ?
(one or more checkboxes)
- yes, I myself have been contaminated
- yes, one of my relatives has been contaminated (family, friend)
- yes, one of my patients has been contaminated.
- yes, one of the patients in the facility where I practice
- no

24.Did you receive psychological support to cope with the professional difficulties related to the Covid-19 crisis?
(1 check box)
- yes, as part of a program that I have benefited from as a mental health services professional
- yes, with a psychiatric and mental health professional, as part of formalized care
- yes, informally with a psychiatric and mental health professional
- no, I have not had psychological support

24.a - If yes
Which ones ?
(open question)

25.What would be your operational recommendations for the psychiatry and mental health sector in your country ?
(open question)

Respondent profile
(drop down list)

Your profession :
- art therapist
- child psychiatrist
- dental surgeon
- dietician
- early childhood educator
- neuropsychologist
- occupational therapist
- physiotherapist
- psychoanalyst
- psychiatric nurse's aide
- psychiatric nurse
- psychiatrist
- psychologist,
- psychotherapist
- social worker
- somatic or general practitioner doctors
- sophrologist
- specialized educator
- speech therapist
- autre : ...

Your specialty :
- addictology

- gerontopsychiatry
- child psychiatry
- forensic psychiatry
- psychiatric emergencies
- as part of a mobile team
- other : …

The context in which you practice :
- within a structure
- as a liberal
- mixed

Your experience :
- in the process of training
- 5≤10
- 11≤20
- ≥21
- ≥30

Your country of exercise during the Covid Pandemic-19 :
- Belgium
- Czech Republic
- Finland
- France
- Germany
- Greece
- Iceland
- Ireland
- Israel
- Italy
- Luxembourg
- Malta
- Poland
- Portugal
- Romania
- Russia
- Spain
- Sweden
- Switzerland
- The Netherlands
- Turkey
- Ukraine
- United Kingdom

Germany :
- Brandenburg
- Berlin
- Bremen
- Baden-Württemberg
- Bavaria
- Hamburg
- Hesse
- Mecklenburg-Western Pomerania

- Lower Saxony
- North Rhine-Westphalia
- Rhineland-Palatinate
- Saarland
- Schleswig-Holstein
- Saxony-Anhalt
- Saxony
- Thuringia

Belgium :
- Antwerp
- Brussels Capital Region
- East Flanders
- Flemish Brabant
- Hainaut
- Liège
- Limburg
- Luxembourg
- Namur
- Walloon Brabant
- West Flanders

Spain :
- Andalusia
- Catalionia
- Community of Madrid
- Valencian Community
- Galicia
- Castile and León
- Basque Country
- Castilla-La-Mancha
- Canary Islands
- Region of Murcia
- Aragon
- Extremadura
- Balearic Islands
- Principality of Asturias
- Chartered Community of Navarre
- Cantabria
- La Rioja

Finland :
- Åland Islands
- Central Finland
- Central Ostrobothnia
- Kainuu
- Kymenlaasko
- Lapland
- North Karelia
- Northern Ostrobothnia
- Northern Savonia
- Ostrobothnia
- Päijänne Tavastia
- Pirkanmaa

- Satakunta
- South Karelia
- Southern Ostrobothnia
- Southern Savonia
- Southwest Finland
- Tavastia Proper
- Uusimaa

France :
- Auvergne-Rhône-Alpes
- Bourgogne-Franche-Comté
- Brittany
- Centre-Val de Loire
- Corsica
- French Guiana
- Grand Est
- Guadeloupe
- Hauts-de-France
- Île-de-France
- Martinique
- Mayotte
- Normandy
- Nouvelle-Aquitaine
- Occitanie
- Pays de la Loire
- Provence-Alpes-Côte d'Azur
- Réunion

Grece :
- Attica
- Central Greece
- Central Macedonia
- Crete
- Eastern Macedonia and Thrace
- Epirus
- Ionian Islands
- North Aegean
- Peloponnese
- South Aegean
- Thessaly
- Western Greece
- Western Macedonia
- Monastic Republic of Mount Athos

Irland :
- County of Carlow
- County of Cavan
- County of Clare
- County of Cork
- County of Donegal
- County of Dublin
- County of Galway
- County of Kerry
- County of Kildare

- County of Kilkenny
- King's County
- County of Leitrim
- County of Limerick
- County of Longford
- County of Louth
- County of Mayo
- County of Meath
- County of Monaghan
- Queen's County
- County of Roscommon
- County of Sligo
- County of Tipperary, North Riding
- County of Tipperary, South Riding
- County of Waterford
- County of Westmeath
- County of Wexford
- County of Wicklow

Island :

- Capital Region
- Southern Peninsula
- Western Region
- Westfjords
- Northwestern Region
- Northeastern Region
- Eastern Region
- Southern Region

Israel :

- Judea and Samaria Area
- Central District
- Haifa District
- Jerusalem District
- Northern District
- Southern District
- Tel Aviv District

Italy :

- Abruzzo
- Aosta Valley
- Apulia
- Basilicata
- Calabria
- Campania
- Emilia-Romagna
- Friuli Venezia Giulia
- Lazio
- Liguria
- Lombardy
- Marche
- Molise
- Piedmont
- Sardinia

- Sicily
- Trentino-South Tyrol
- Tuscany
- Umbria
- Veneto

Luxembourg :

- Diekirch
- Grevenmacher
- Luxembourg

Malta :

- Central region
- Gozo Region
- Northern Region
- South Eastern region
- Southern Region

Czech Republic :

- Central Bohemian Region
- Hradec Králové Region
- Karlovy Vary Region
- Moravian-Silesian Region
- Liberec Region
- Olomouc Region
- Pardubice Region
- Plzeň Region
- Prague
- South Bohemian Region
- South Moravian Region
- Ústí nad Labem Region
- Vysočina Region
- Zlín Region

Netherlands:

- Drenthe
- Flevoland
- Friesland
- Gelderland
- Groningen
- Limburg
- Noord-Brabant
- Noord-Holland
- Overijssel
- Zuid-Holland
- Utrecht
- Zeeland

Poland :

- Greater Poland
- Kuyavia-Pomerania
- Lesser Poland
- Łódź

- Lower Silesia
- Lublin
- Lubusz
- Masovia
- Opole
- Podlaskie
- Pomerania
- Silesia
- Subcarpathia
- Holy Cross Province
- Warmia-Masuria
- West Pomerania

Portugal :
- Aveiro
- Beja
- Braga
- Bragança
- Castelo Branco
- Coimbra
- Évora
- Faro
- Guarda
- Leiria
- Lisbon
- Portalegre
- Porto
- Santarém
- Setúbal
- Viana do Castelo
- Vila Real
- Viseu

Romania :
- Bucuresti-Ilfox development region
- Centru development region
- Nord-Est development region
- Nord-Vest development region
- Sud-Est development region
- Sud-Muntenia development region
- Sud-Vest Oltenia development region
- Vest development region

United Kingdom :
- East of England
- East Midlands
- London
- North East
- North West
- South East
- South West
- West Midlands
- Yorkshire and the Humber

Russia :
- Central
- Central Black Earth
- East Siberian
- Far Eastern
- Kaliningrad
- North Caucasus
- Northern
- Northwestern
- Ural
- Volga
- Volga-Vyatka
- West Siberian

Sweden :
- Blekinge
- Dalarna
- Gävleborg
- Gotland
- Halland
- Jämtland
- Jönköping
- Kalmar
- Kronoberg
- Norrbotten
- Örebro
- Östergötland
- Skåne
- Södermanland
- Stockholm
- Uppsala
- Värmland
- Västerbotten
- Västernorrland
- Västmanland
- Västra Götaland

Switzerland :
- Aargau
- Appenzell Ausserrhoden
- Appenzell Innerrhoden
- Basel-City
- Basel-Country
- Berne
- Fribourg
- Geneva
- Glarus
- Graubünden
- Jura
- Lucerne
- Neuchâtel
- Nidwalden
- Obwalden
- Schaffhausen

- Schwyz
- Solothurn
- St. Gallen
- Thurgau
- Ticino
- Uri
- Valais
- Vaud
- Zug
- Zürich

Turkey :
- Aegean
- Blacksea
- Central Anatolia
- Eastern Anatolia
- Marmara
- Mediterranean
- Southeastern Anatolia

Ukraine :
- Autonomous Republic of Crimea
- Kyiv
- Sevastopol
- Oblast de Tcherkassy
- Oblast de Tchernihiv
- Oblast de Tchernivtsi
- Oblast de Dnipropetrovsk
- Oblast de Donetsk
- Oblast d'Ivano-Frankivsk
- Oblast de Kharkiv
- Oblast de Kherson
- Oblast de Khmelnitski
- Oblast de Kirovohrad
- Oblast de Kiev
- Oblast de Louhansk
- Oblast de Lviv
- Oblast de Mykolaïv
- Oblast d'Odessa
- Oblast de Poltava
- Oblast de Rivne
- Oblast de Soumy
- Oblast de Ternopil
- Oblast de Vinnytsia
- Oblast de Volhynie
- Oblast de Transcarpatie
- Oblast de Zaporijia
- Oblast de Jytomyr

You are:
- 18-25
- 26-55
- 56-70

You are:

- man
- woman
- other

GHQ

Recently

1. Have you been able to concentrate on everything you do?

better than usual
as usual
less well than usual
much less well than usual

2. Have you lacked sleep because of your worries?

not at all
no more than usual
a little more than usual
much more than usual

3. Did you feel able to make decisions?

more than usual
as usual
less well than usual
much less well than usual

4. Did you feel constantly tense or "stressed"?

not at all
no more than usual
a little more than usual
much more than usual

5. Did you feel that you played a useful role in life?

more than usual
as usual
less useful than usual
much less useful than usual

6. Did you feel that you could not overcome your difficulties?

not at all
no more than usual
a little more than usual
much more than usual

7. Were you able to enjoy your normal daily activities?

more than usual
as usual
less than usual
much less than usual

8. Were you able to cope with your problems?

better than usual
as usual
a little less than usual
much less than usual

9. Have you been unhappy and depressed?

not at all
no more than usual
a little more than usual
much more than usual

10. Have you lost confidence in yourself?

not at all
no more than usual
a little more than usual
much more than usual

11. Did you consider yourself to be worthless?

not at all
no more than usual
a little more than usual
much more than usual

12. Did you feel reasonably happy, all things considered?

more than usual
as usual
a little less than usual
much less than usual

MHPCI

1. I give users as much information as possible about their diagnosis.

from 0 to 4
0 = never
1 = rarely
2 = sometimes
3 = often
4 = always

2. I maintain regular contact with the family of the users.

from 0 to 4
0 = never
1 = rarely
2 = sometimes
3 = often
4 = always

3. I have the impression that my thoughts about service users are not understood by other colleagues.

from 0 to 4
0 = never
1 = rarely
2 = sometimes
3 = often
4 = always

4. I control myself so as not to show my negative feelings towards the users.

from 0 to 4
0 = never
1 = rarely
2 = sometimes
3 = often
4 = always

5. I can count on the relatives of the users.
from 0 to 4
0 = never
1 = rarely
2 = sometimes
3 = often
4 = always
6. I am the object of threats or aggressive acts by users.
from 0 to 4
0 = never
1 = rarely
2 = sometimes
3 = often
4 = always
7. I talk with users about topics concerning spirituality and religion.
from 0 to 4
0 = never
1 = rarely
2 = sometimes
3 = often
4 = always
8. I help users to accept their homosexuality as an important and positive aspect of their identity. (If no users are of homosexual orientation, answer "0 = never").
from 0 to 4
0 = never
1 = rarely
2 = sometimes
3 = often
4 = always
9. I actively involve those close to the users in my work.
from 0 to 4
0 = never
1 = rarely
2 = sometimes
3 = often
4 = always
10. My work with users is based on clear and well-documented theoretical models.
from 0 to 4
0 = never
1 = rarely
2 = sometimes
3 = often
4 = always
11. I am always on the alert, so as not to be caught off guard by unpredictable aggressive acts on the part of users.
from 0 to 4
0 = never
1 = rarely
2 = sometimes
3 = often
4 = always
12. I find it difficult to intervene effectively when users have religious beliefs.
from 0 to 4
0 = never

1 = rarely
2 = sometimes
3 = often
4 = always

13. I give my (personal) telephone number to my users so that they can reach me if necessary.

from 0 to 4
0 = never
1 = rarely
2 = sometimes
3 = often
4 = always

14. I easily manage aggressive behaviour by users. (If users do not behave aggressively, answer "0 = never").

from 0 to 4
0 = never
1 = rarely
2 = sometimes
3 = often
4 = always

15. I talk with users about my thoughts, feelings and experiences.

from 0 to 4
0 = never
1 = rarely
2 = sometimes
3 = often
4 = always

16. It seems to me that there are other colleagues who do not have the same training or profession as me, yet do the same work as me.

from 0 to 4
0 = never
1 = rarely
2 = sometimes
3 = often
4 = always

17. I am involved in the legal and judicial aspects of my users.

from 0 to 4
0 = never
1 = rarely
2 = sometimes
3 = often
4 = always

18. I use a learning system with users based on rewards and punishments.

from 0 to 4
0 = never
1 = rarely
2 = sometimes
3 = often
4 = always

19. I avoid discussing the topic of sexuality with users because it bothers them.

from 0 to 4
0 = never
1 = rarely
2 = sometimes
3 = often

4 = always

20. I apologise to the user if I realise that I was wrong.

from 0 to 4
0 = never
1 = rarely
2 = sometimes
3 = often
4 = always

21. Users contact me outside working hours.

from 0 to 4
0 = never
1 = rarely
2 = sometimes
3 = often
4 = always

22. I manage to integrate protocols or intervention manuals with my way of working.

from 0 to 4
0 = never
1 = rarely
2 = sometimes
3 = often
4 = always

23. I communicate with users via the Internet.

from 0 to 4
0 = never
1 = rarely
2 = sometimes
3 = often
4 = always

24. I take into account the opinions of workers from other services (mental health or social services) who are in contact with the users I meet, whatever their opinion.

from 0 to 4
0 = never
1 = rarely
2 = sometimes
3 = often
4 = always

25. I dedicate part of my time to keeping in touch with workers from other services who follow my users.

from 0 to 4
0 = never
1 = rarely
2 = sometimes
3 = often
4 = always

26. I insist that users respect the rules and limits.

from 0 to 4
0 = never
1 = rarely
2 = sometimes
3 = often
4 = always

27. I get upset if a colleague gets angry with a user.

from 0 to 4

0 = never
1 = rarely
2 = sometimes
3 = often
4 = always

28. I think that the aggressive acts of users are the result of intense states of distress that they cannot control (if users do not have aggressive behaviour, answer "0 = never").

from 0 to 4
0 = never
1 = rarely
2 = sometimes
3 = often
4 = always

29. I feel uncomfortable when a user treats me as if I were their friend.

from 0 to 4
0 = never
1 = rarely
2 = sometimes
3 = often
4 = always

30. I establish with the users the rules we agree to respect.

from 0 to 4
0 = never
1 = rarely
2 = sometimes
3 = often
4 = always

31. In my work, I can only be effective if I define clear and concrete objectives.

from 0 to 4
0 = never
1 = rarely
2 = sometimes
3 = often
4 = always

32. I agree to hear users talk about their sexuality.

from 0 to 4
0 = never
1 = rarely
2 = sometimes
3 = often
4 = always

33. I find it difficult to understand the professional language used by other professionals who follow my clients.

from 0 to 4
0 = never
1 = rarely
2 = sometimes
3 = often
4 = always

34. I try to keep up to date with the techniques deemed most effective by the scientific community.

from 0 to 4
0 = never
1 = rarely

2 = sometimes
3 = often
4 = always

35. I avoid having a friendly approach with users.

from 0 to 4
0 = never
1 = rarely
2 = sometimes
3 = often
4 = always

36. I avoid being alone with users with a severe mental disorder (if the users do not have a severe mental disorder, answer "0 = never").

from 0 to 4
0 = never
1 = rarely
2 = sometimes
3 = often
4 = always

37. I have noticed that the fact that the user is a believer/believer in a religion has a positive influence on his mental health status.

from 0 to 4
0 = never
1 = rarely
2 = sometimes
3 = often
4 = always

38. Working with users creates overload and emotional exhaustion for me.

from 0 to 4
0 = never
1 = rarely
2 = sometimes
3 = often
4 = always

39. When I discuss a user with my colleagues, I also talk about their sex life.

from 0 to 4
0 = never
1 = rarely
2 = sometimes
3 = often
4 = always

40. In my work, I make contact with friends, neighbours or colleagues of users.

from 0 to 4
0 = never
1 = rarely
2 = sometimes
3 = often
4 = always

41. I am upset by the requests for attention from users.

from 0 to 4
0 = never
1 = rarely
2 = sometimes
3 = often
4 = always

42. My colleagues ask me for my views on users, drawing on the specific skills of my profession.

from 0 to 4
0 = never
1 = rarely
2 = sometimes
3 = often
4 = always

43. The advice of other clinicians who have followed the client in the past helps me to establish therapeutic interventions or rehabilitation activities.

from 0 to 4
0 = never
1 = rarely
2 = sometimes
3 = often
4 = always

44. When I discuss a user with my colleagues, I also talk about his or her spiritual life.

from 0 to 4
0 = never
1 = rarely
2 = sometimes
3 = often
4 = always

45. I can really improve a user's quality of life if I take into account their family and social context.

from 0 to 4
0 = never
1 = rarely
2 = sometimes
3 = often
4 = always

46. I use scales, questionnaires or tests to assess change among users.

from 0 to 4
0 = never
1 = rarely
2 = sometimes
3 = often
4 = always

47. The behaviour of the users makes me lose patience.

from 0 to 4
0 = never
1 = rarely
2 = sometimes
3 = often
4 = always

48. What I say to a user is denied by a colleague.

from 0 to 4
0 = never
1 = rarely
2 = sometimes
3 = often
4 = always

49. Sometimes I talk to users about topics such as masturbation, sexual intercourse and the use of contraceptives.

from 0 to 4
0 = never
1 = rarely
2 = sometimes
3 = often
4 = always
