# Supplementary material for: International professional practices in mental health, organization of psychiatric care, and COVID-19: A survey protocol
Source: PLoS One. 2021 Dec 28;16(12):e0261818. doi: 10.1371/journal.pone.0261818 (PMC8714114; doi:10.1371/journal.pone.0261818)
Supplement: Attachment — Submitted filename: Response to Reviewers.docx [file pone.0261818.s002.docx]

Response to Reviewers

Reviewer #1: PlosOne Manuscript Number PONE-D-21-09107

Title: “International professional practices in mental health organization of psychiatric care, and Covid-19: A survey protocol

Overview:

This study protocol aims to investigate the impact of the Covid-19 pandemic on mental health care providers/professionals using survey data from 2,000 individuals located across 23 countries. The data collected will be used to develop tools to improve mental health care delivery during a global health crisis, as well as specific monitoring and support tools for mental health professionals. Overall, the proposed work addresses an important topic, as the world looks to recover from this pandemic and prepare for future public health crises. The writing and presentation, however, make it very unclear what has already been done, versus what aspects of this study have yet to be completed.

Specific comments:

Introduction-

1. In my opinion, too much time is spent discussing the impact of COVID-19 pandemic on the health care community in general, as this has been well described in the academic literature as well as the popular media. The focus here is more specifically mental health care providers and organizations.

Response:

As requested, we have revised the "introduction" section to focus more on mental health care providers and organizations. We also revised the “references” section.

Methods-

1. Since a standard questionnaire was not used, access to the full final survey should be provided as a supplement, or at least specific categories and examples of questions from each category. I see that there is an included supplemental material file, but it is not referenced in the body of the manuscript, so it is rather unclear what this table represents.

Response:

The study questionnaire (Additional file 1) aims to investigate the following themes:

- Local organizational adaptations during the pandemic for the continuation of care in compliance with health and ethical conditions;

- The modalities of use of telepsychiatry and online mental health monitoring and the impact of the use of these tools in interprofessional relations and in the care relationship;

- The impact of COVID-19 on working conditions in psychiatry and on the mental health of professionals in this sector. We have added this paragraph to line 143.

2. Figure 1- What happened to M7? Is there a more specific timeline (April 2021-March 2022, for example)?

Response:

Thank you for pointing this mistake. M7 corresponds to the time spent validating the survey tools with the scientific committee. We have modified Figure 1 to clarify this point.

3. Figure 2- this figure is not necessary; add a column to Table 1 that includes the actual number of HPP included in the survey.

Response:

We agree that the information given in Figure 2 is redundant with that in Table 1 in the column entitled: Number of selected HPPs* per country. As suggested, we have removed Figure 1.

4. Given the international nature of this study, what language will/has been used for data collection and data management?

Response:

The initial version of the questionnaire will be designed in French, and this version will be distributed in some countries including France. The questionnaire has been translated into the official language(s) of the different countries involved in the study: English, German, Portuguese, Spanish, Finnish, Greek, Hebrew, Hindi, Italian, Dutch, Polish, Romanian, Russian and Ukrainian. The questionnaire is also available to all respondents in English.We have added this sentence Page 9, line 190.

We thank the reviewers for giving us an opportunity to substantially improve the content and the presentation of our manuscript.
