# Supplementary material for: International professional practices in mental health, organization of psychiatric care, and COVID-19: A survey protocol
Source: PLoS One. 2021 Dec 28;16(12):e0261818. doi: 10.1371/journal.pone.0261818 (PMC8714114; doi:10.1371/journal.pone.0261818)
Supplement: Attachment — Submitted filename: Response to reviewers.docx [file pone.0261818.s003.docx]

Editor comments:

The manuscript requires English Proof.

Response:

The English version of this manuscript was corrected by the “American Manuscript Editors”. An "English Editing Certificate" was associated with the submission of this revised version.

Reviewer #1: Summary:

This work aims to build on preliminary work to better understand the psychological and societal impacts of the Covid-19 pandemic on psychiatry professionals and mental health services/organizations in order to inform best practices regarding psychiatric support and recovery during this, and future public health crises. While the study and proposed work address important questions, the writing lacks clarity and is full of typos, making it difficult for this reviewer to fully assess the scientific content of this manuscript.

Response:

We thank the reviewer for giving us an opportunity to substantially improve the content and the presentation of our manuscript with constructive comments.

Introduction

1. The final sentence of the first paragraph (lines 9-12) is unclear (and appears to have a missing word, or typo?). Furthermore, how can the effects have already lasted “several years after the trauma” when the pandemic was declared less than 2 years ago:

They exhibited signs of anger, anxiety, and stress related to the risk of contamination and the uncertainty in daily routines [3] and lasting negative effects several years after the trauma like include symptoms related to alcohol consumption, insomnia, and depression [3-5].

Response:

To introduce this paragraph, we have given some general information on Sars-CoV2. Without precise knowledge of the consequences of this infection, we have compared it to the SARS epidemic in order to highlight certain elements already known about the working conditions of health professionals in such a context.

We have made some corrections using the track changes, lines 2 to 12. We hope this paragraph will be clearer

Study Design and Methods

2. Please provide clarification regarding the difference between “questionnaire grids” versus “themes” in the text. For example, are each of the themes being examined in each grid?

Response:

The aim of qualitative research is to develop concepts that help us understand social phenomena in natural (rather than experimental) contexts, focusing on the meanings, experiences and perspectives of all the different participants in the study. The analysis of this information allows themes to emerge that will be further analysed through different techniques such as semi-structured individual interviews and focus groups (with questionnaire grids to explore all the selected themes).

In the “First step: Questionnaire grids creation” section we have added the sentence below.

“*These exploratory interviews will allow the description of behaviors, situations and emotions in real life situations.”*

Line 125 for clarification we have deleted "results" and replaced it with "themes".

3. Please explain what is meant by “20 focus groups of mental health professionals.”

Response:

This survey protocol (focus groups) makes it possible to collect the opinions of several people. This technique also makes it possible to study the social relations between the people present. A number of 20 persons is needed for data saturation.

We have added a reference to justify this point lines 133 and 146.

[14]. Marshall B, Cardon P, Poddar A, Fontenot R. Does Sample Size Matter in Qualitative Research? A Review of Qualitative Interviews in this Research. Journal of computer information systems.2013; 54(1), 11-22.

And listed it in the reference section.

4. The terms inside parentheses on lines 137-138: What is this in referencing? For example, is this initial phase meant to address the feasibility of delivering the questionnaire, in terms of duration/time to deliver and complete, clarity of the questions, etc.?

Response:

To clarified this point, we deleted the sentence in parentheses and added line 146 “The elements evaluated will be the time taken to complete the questionnaire and the good understanding of the questions by the participants.”

5. Figure 1: Please do not use acronyms/abbreviations in the figure title, especially if they have not been introduced in the text. Please check flow chart text for typos and standardize the formatting/organization of the text; perhaps consider dividing each box into explicit "actions", "sub-tasks/actions" and "outcomes/aims"

Response:

Thanks for the comment. Line 89, we introduced the acronym of our study: Psy-GIPOC.

As requested, we checked flow chart text for typos and standardize the formatting/organization of the text. We thank the reviewer for suggesting that each box be divided into explicit "actions", "subtasks/actions" and "outcomes/objectives".

Reviewer #2: Authors of article entitled "International professional practices in mental health, organization of psychiatric care, and Covid-19: A survey protocol" have addressed suggested comments.

Response:

Thank you
